# Publisher Correction: Imaging spectroscopy of solar radio burst fine structures

**DOI:** 10.1038/s41467-017-02464-6

**Published:** 2018-01-11

**Authors:** E. P. Kontar, S. Yu, A. A. Kuznetsov, A. G. Emslie, B. Alcock, N. L. S. Jeffrey, V. N. Melnik, N. H. Bian, P. Subramanian

**Affiliations:** 1 School of Physics and Astronomy, University of Glasgow, Glasgow, G12 8QQ UK; 2 New Jersey Institute of Technology, Newark, NJ 07102 USA; 3 Key Laboratory of Solar Activity, National Astronomical Observatories, Chinese Academy of Sciences, Beijing, 100012 China; 4 Institute of Solar-Terrestrial Physics, Irkutsk, 664033 Russia; 5 Department of Physics & Astronomy, Western Kentucky University, Bowling Green, Kentucky, 42101 USA; 6 Institute of Radio Astronomy, National Academy of Sciences of Ukraine, Kharkiv, 61002 Ukraine; 7 Indian Institute of Science Education and Research, Pune, 411008 India

Correction to: *Nature Communications* 10.1038/s41467-017-01307-8, published online 15 November 2017

The original version of this article contained errors in Refs 15, 27, 32, 33 and 43, which were incorrectly given with the wrong journal name “*Solid Phys*.” rather than the correct “*Sol. Phys*.”. This has now been corrected in the PDF and HTML versions of the article.

