# Supplementary material for: Imaging spectroscopy of solar radio burst fine structures
Source: Nat Commun. 2017 Nov 15;8:1515. doi: 10.1038/s41467-017-01307-8 (PMC5688146; doi:10.1038/s41467-017-01307-8)
Supplement: Supplementary file 1 — Description of Additional Supplementary Files [file 41467_2017_1307_MOESM1_ESM.pdf]

Description of Additional Supplementary Files

File Name: Supplementary Movie 1
Description: **Low Frequency Array (LOFAR) animated images at the selected 32 MHz frequency**. The Full Width at Half-Maximum (FWHM) ellipses made using two-dimensional Gaussian fits to the data are shown in blue. The white crosses show the phased array beam locations and the oval show half-maximum synthesised LOFAR beam.
